# Motives for MDMA Use: A Comparative Study with Alcohol and Cannabis

**DOI:** 10.1192/j.eurpsy.2024.864

**Published:** 2024-08-27

**Authors:** D. Zullino, L. Penzenstadler, S. Rothen, F. Seragnnoli, A. S. Girardet, G. Calzada, G. Thorens

**Affiliations:** ^1^ Service d’addictologie; ^2^Hôpitaux Universitaires de Genève, Genève, Switzerland

## Abstract

**Introduction:**

While research on drug use motives has primarily focused on widely used substances like alcohol, tobacco, and cannabis, understanding the motivations behind MDMA use is crucial for developing targeted therapeutic, preventive, and harm reduction strategies.

**Objectives:**

The objective of this study is to present the findings of an online survey that evaluates the motives behind the use of 3,4-methylenedioxymethamphetamine (MDMA) and draws comparisons between these motives and those for alcohol and cannabis consumption.

**Methods:**

Data were collected through an online survey, available in both English and French, with 99 participants. The survey included five sections, including a substance abuse screening test (ASSIST) and the Pahnke-Richards Mystical Experience Questionnaire. The primary focus was on motives for MDMA use, assessed using an adapted version of the Marijuana Motives Measure (MMM), comparing them with alcohol and cannabis motives.

**Results:**

The most reported motive for MDMA use was enhancement, followed by expansion motives. Social motives were the third most common, while coping motives ranked fourth, and conformity was the least common motive. Comparisons with alcohol and cannabis use motives revealed differences in motives for each substance. MDMA showed a unique pattern of motives.

**Conclusions:**

Enhancement emerged as the most prevalent motive for MDMA use, consistent with previous research on MDMA motive use. Expansion motives, which involve altering perceptions and increasing self-awareness, ranked second, reflecting the growing interest in MDMA-assisted therapy for conditions like PTSD. Surprisingly, social motives were less common for MDMA compared to alcohol and cannabis, suggesting unique social dynamics associated with MDMA use. Conformity motives were also less significant for MDMA users, possibly due to the age of initiation and user maturity.

Understanding the motives behind MDMA use is essential for designing effective interventions and harm reduction strategies. The distinct motives for MDMA, as compared to alcohol and cannabis, highlight the need for tailored approaches to address its use. Further research should explore the complex interplay of motives, age of initiation, social context, and cultural factors to inform comprehensive strategies related to MDMA use.

**Disclosure of Interest:**

None Declared

